# Transcriptional insights into gastrointestinal adaptations in pigs to high altitude

**DOI:** 10.3389/fvets.2025.1723710

**Published:** 2025-12-04

**Authors:** Bo Ran, Jie Tang, Yan Wang, Xuan Tao, Yuekui Yang, Xuemei Yang, Jianjun Gong, Zhiping He, Yiren Gu, Pengliang Liu, Yan Liang

**Affiliations:** 1 Animal Genetic Breeding and Reproduction Key Laboratory of Sichuan Province, Sichuan Animal Science Academy, Chengdu, China; 2 Key Laboratory of Qinghai-Tibetan Plateau Animal Genetic Resource Reservation and Utilization, Ministry of Education, Southwest Minzu University, Chengdu, China

**Keywords:** Tibetan pigs, gastrointestinal tract, gene expression, high-altitude adaptation, skeletal muscle

## Abstract

Characterizing the transcriptomic profiles of gastrointestinal tract tissues in high-altitude pigs is crucial for understanding the molecular mechanisms underlying their metabolic adaptation to high-altitude environments. Here, we generated RNA-seq data from five gastrointestinal tract tissues (i.e., stomach, jejunum, cecum, colon, rectum) and the triceps brachii muscle of six 300-day-old castrated male Tibetan pigs (inhabiting high-altitude regions) and six Black pigs (inhabiting low-altitude regions). Pigs were group-housed and fasted for 24 h before sampling. We identified transcriptional differences between these two pig populations, revealing that genes upregulated in the gastrointestinal tract tissues and triceps brachii muscle of Tibetan pigs, compared to Black pigs, are primarily associated with immune response and metabolic processes, including lipid metabolism. Consistently, a comparative analysis demonstrated that the fatty acid content was higher in the triceps brachii muscles of Tibetan pigs than in those of Black pigs. Additionally, we identified 18 genes, including *HDC*, *SETD9*, *HUS1*, and *RPSA*, whose expression in gastrointestinal tract tissues was significantly correlated with the metabolite abundance (amino acid and fatty acid, etc.) in the triceps brachii muscles. This study may contribute to the understanding of high-altitude adaptation mechanisms in Tibetan pigs and provides valuable insights for further genetic improvement of pig breeds.

## 1 Introduction

The Tibetan pig predominantly resides in the high-altitude regions (2,500–4,300 m) of the Qinghai–Tibet Plateau in China and exhibits significant adaptation to extreme elevations (1). As such, it serves as an ideal animal model for studying the molecular mechanisms underlying high-altitude adaptation. Comparative genomic analysis between Tibetan pigs and low-altitude pigs has identified several rapidly evolving and positively selected genes in Tibetan pigs, many of which are associated with energy metabolism (2, 3). For example, Li et al. identified 21 genes associated with energy metabolism that underwent positive selection in Tibetan pigs compared to Duroc pigs (2). Through a comparative analysis of genetic differences between Tibetan pigs and various lowland pig breeds, Yang et al. discovered that multiple genes under selection in Tibetan pigs are implicated in energy metabolism (4). Additionally, the energy metabolism-related positively selected genes have also been reported in other animals inhabiting high-altitude regions (5–7), underscoring the significance of energy metabolism in high-altitude adaptation. However, the potential mechanisms of metabolic adaptation in high-altitude remain to be elucidated.

The mammalian gastrointestinal tract is a crucial organ responsible for nutrient digestion and absorption, and it plays an important role in energy metabolism. In addition, the gastrointestinal tract also participates in the metabolic regulation of tissues with high metabolic activity, such as skeletal muscle and liver (8–10). Recent studies have documented the adaptation of gut microbiota to high-altitude environments in mammals (11–13). For instance, studies on the microbiome of Tibetan pigs have demonstrated the critical role of gastrointestinal microbiota in facilitating their adaptation to high-altitude conditions. This adaptation is achieved through the optimization of carbohydrate metabolism and the production of short- and medium-chain fatty acids, thereby enhancing energy utilization efficiency (14, 15). However, the adaptive modifications of the gastrointestinal tract itself in response to high-altitude conditions, as well as its effects on other metabolic tissues, remain inadequately explored.

In this study, we generated transcriptomic data for five gastrointestinal sites (including the stomach, jejunum, cecum, colon, and rectum) as well as the triceps brachii muscle in Tibetan pigs and Black pigs (a pig population residing at low altitude). We analyzed the transcriptional differences between these two pig populations and investigated the potential associations between gene expression in various gastrointestinal sites and metabolite abundance (amino acids, fatty acids, etc.) in the triceps brachii. Our findings enhance the understanding of the functional divergence of gastrointestinal tissues between high- and low-altitude pigs and provide valuable resources for future research on high-altitude adaptation in livestock.

## 2 Materials and methods

### 2.1 Animals and sample preparation

In this study, pig samples were collected from two populations, the healthy 300-day-old castrated male plain Black pig (*n* = 6, 461 m, from Suining, Sichuan, China) and the Tibetan pig (*n* = 6, 3,750 m, from Daocheng, Sichuan, China). The pigs were housed in groups (~3 m^2^/pig) and were fed under the same feeding conditions. They were fed twice daily with formula diets (digestible energy 12.56 MJ/kg, crude protein 15%, Lysine 0.8%, calcium 0.7%, phosphorus 0.6%) and had ad libitum access to water. The pigs were humanely sacrificed after fasting for 24 h. A total of 12 triceps brachii samples were collected (6 samples per population) from each pig for the measurement of amino acids, fatty acids, and flavor substances and for transcriptome sequencing. Furthermore, a total of 60 gastrointestinal tract tissue samples were collected for transcriptome sequencing. Of these, 59 samples were successfully sequenced. One colon sample from Black pigs was excluded from the analysis post-sequencing due to insufficient quality (Supplementary Table S1). These samples were from five different parts of the gastrointestinal tract: stomach, jejunum, cecum, colon, and rectum. The collected samples were quickly transferred onto dry ice for transportation, and were finally stored in a − 80 °C freezer.

### 2.2 Determination of amino acid content

A total of 12 triceps brachii samples were used (6 samples per population) to determine the amino acid content. The amino acid analysis was conducted following the method described in a previous study with minor modifications (16). Briefly, approximately 1 g of sample was weighed and placed at the bottom of a specialized hydrolysis tube, to which 8 mL of 6 mol/L hydrochloric acid (HCl) solution was slowly added. The hydrolysis tube was gently rotated to ensure the complete wetting of the sample. The system was then evacuated, maintained under vacuum for 5 min, sealed, and hydrolyzed at 110 ± 1 °C for 22–24 h. Upon the completion of hydrolysis, the tube was cut open, and the contents transferred to a 25 mL volumetric flask, using deionized water to achieve complete transfer. The solution was then adjusted to the final volume (25 mL) and filtered through double-layered filter paper. An aliquot (1 mL) of the filtrate was transferred to a 25 mL beaker and evaporated to dryness in a vacuum desiccator (water bath temperature not exceeding 50 °C). Hydrochloric acid (1 mL) with a pH of 2.2 was added for dissolution. The resulting solution was then transferred to a 1.5 mL centrifuge tube and the sample centrifuged at 10,000 rpm for 10 min. An aliquot (0.5 mL) of the supernatant was placed in a sample vial for analysis. The amino acid contents were measured with an Agilent liquid chromatograph (model: Agilent 1,100). A total of 14 amino acid contents were measured, including 7 essential amino acids and 7 non-essential amino acids.

### 2.3 Determination of fatty acid content

A total of 12 triceps brachii samples were used (*n* = 6 for each population) to determine the fatty acid content. The fatty acid content analysis was conducted following the method described in a previous report with minor modifications (17). Briefly, samples (1.0 g) were subjected to ultrasonic extraction with a methanol–chloroform mixture (2:1), followed by filtration. The filtrate was concentrated under nitrogen evaporation, and then dissolved in a solution of ether and hexane (2:1). To this solution was added 1 M KOH–methanol, and the mixture was reacted in a water bath at 70 °C for 30 min. Upon cooling, 1 mL of deionized water was added to facilitate phase separation. The upper layer was then collected for analysis. A DB-WAX column (30 m × 0.25 mm × 0.25 μm) was used for gas chromatography–mass spectrometry (GC–MS) analyses. Helium was used as the carrier gas at a flow rate of 1 mL/min. The injector temperature was set at 250 °C with a split ratio of 10:1. The initial temperature was held at 170 °C for 1 min, then ramped at 5 °C/min to 230 °C, where it was held for 15 min. The transfer line temperature was maintained at 250 °C. The ion source type was EI+, with an ion source temperature of 200 °C. The electron impact energy was set to 70 eV, and the emission current was 50 μA. The mass scan range was from 33 to 450 amu. Spectral library searches were conducted with the NIST 2005 and Wiley 7 libraries.

### 2.4 Determination of volatile flavor compounds

A total of 12 triceps brachii samples were used (*n* = 6 for each population) to determine the content of flavor substances. The detection of flavor substances was carried out following the method outlined in a previous paper with minor modifications (18). Briefly, for solid-phase microextraction (SPME), each sample of porcine triceps brachii muscle was minced into particles of approximately 2 mm in size and weighing 6 g, which were then placed into a 15 mL sample vial. The samples were extracted at 70 °C for 30 min. The extraction fiber used was *divinylbenzene/carboxen/polydimethylsiloxane* with a coating thickness of 50/30 mm and a fiber length of 2 cm. Extraction was performed at 70 °C for 40 min. The desorption temperature was 250 °C and the desorption time was 3 min. The fiber was conditioned according to the manufacturer’s recommendations before its first use in the gas chromatograph. The analysis was performed with a SCION SQ 456-GC gas chromatograph–mass spectrometer (Bruker Corporation, United States). The injection port temperature was set at 250 °C with a non-split injection mode. Helium was used as the carrier gas, flowing at a rate of 1 mL/min. A DB-WAX chromatographic column was used, measuring 30 m (length) × 0.25 mm (inner diameter) × 0.25 μm (film thickness). The temperature program was initiated at 40 °C, which was maintained for 3 min, ramped to 90 °C at 5 °C/min, then further ramped to 230 °C at 10 °C/min, and held at 230 °C for 7 min. The MS analysis was conducted as described for the determination of the content of 13 volatile flavor fatty acids in total.

### 2.5 Transcriptome analysis

A total of 59 gastrointestinal tract tissue samples were utilized for transcriptome sequencing. Total RNA was extracted from each sample using the RNeasy Mini Kit (Qiagen, Valencia, CA, United States). The purified RNA was quantified with a Nanodrop spectrophotometer, and its integrity was evaluated using an Agilent 2000 bioanalyzer. The mRNA was isolated from the total RNA using oligo (dT) magnetic beads. The isolated mRNA was fragmented using a fragmentation buffer to produce short fragments. First-strand cDNA synthesis was performed using random primers, followed by the synthesis of double-stranded cDNA. The resulting cDNA underwent end-repair and 3′ adenylation. Adaptors were ligated to the 3′ adenylated cDNA fragments. The adaptor-ligated products were then amplified by PCR. The final library was obtained following quality control and product cyclization. All libraries were sequenced on the DNBSEQ platform (BGI) with a paired-end read length of 150 bp.

The high-quality reads obtained were mapped to the pig reference genome (Sscrofa11.1) with STAR v2.7.6a (19). The pig reference genome and the corresponding gene annotation were downloaded from the Ensembl database. The expression levels of transcripts were quantified as transcripts per million (TPM) values with Kallisto v0.44.0 (20). The differential expression of the genes was analyzed with DESeq2 v1.28.1 (21). Genes were considered differentially expressed when FDR < 0.05 (Benjamini–Hochberg correction) and fold change>2. Functional enrichment analysis of specific gene sets was conducted utilizing Metascape (22, 23) with its default parameters. Gene Ontology Biological Processes (GO-BP) and Kyoto Encyclopedia of Genes and Genomes (KEGG) pathways were chosen as the ontology sources. The most significant term within each cluster of the enrichment results is presented.

### 2.6 Real-time quantitative PCR

First-strand cDNA was synthesized from 1 μg of total RNA using the PrimeScript RT reagent Kit with gDNA Eraser (TaKaRa, Japan). qPCR was performed on a LightCycler 480 II Real-Time PCR System (Roche, Switzerland) using SYBR Green Premix Pro Taq HS (Accurate Biology, China). Each reaction was carried out in a 20 μL volume containing 10 μL of 2 × SYBR Green Master Mix, 0.4 μL of each forward and reverse primer (10 μM), 2 μL of diluted cDNA template, and 7.2 μL of nuclease-free water. The thermal cycling conditions were as follows: initial denaturation at 95 °C for 30 s; followed by 40 cycles of 95 °C for 5 s and 60 °C for 30 s. A melt curve analysis was performed at the end of each run (95 °C for 15 s, 60 °C for 60 s, and 95 °C for 15 s) to confirm the specificity of the amplification and the absence of primer-dimers or non-specific products. All reactions, including no-template controls (NTCs), were run in triplicate. Gene-specific primers for the target gene (*HDC*) and the reference gene (*GAPDH*) were designed using Primer-BLAST (NCBI) and synthesized by Tsingke Biotechnology (China) (Supplementary Table S25). The relative expression level of each target gene was calculated using the comparative 2^(-ΔΔCt) method (24). The stable expression of the *GAPDH* gene was used for normalization. The group of Black pigs was designated as the calibrator (set to 1). Statistical significance between the Tibetan and Black pig groups for each gene and tissue was determined using an unpaired, two-tailed Student’s t-test.

## 3 Results and discussion

### 3.1 Transcriptional characteristics in five gastrointestinal tract tissues of Black and Tibetan pigs

**Figure 1:**
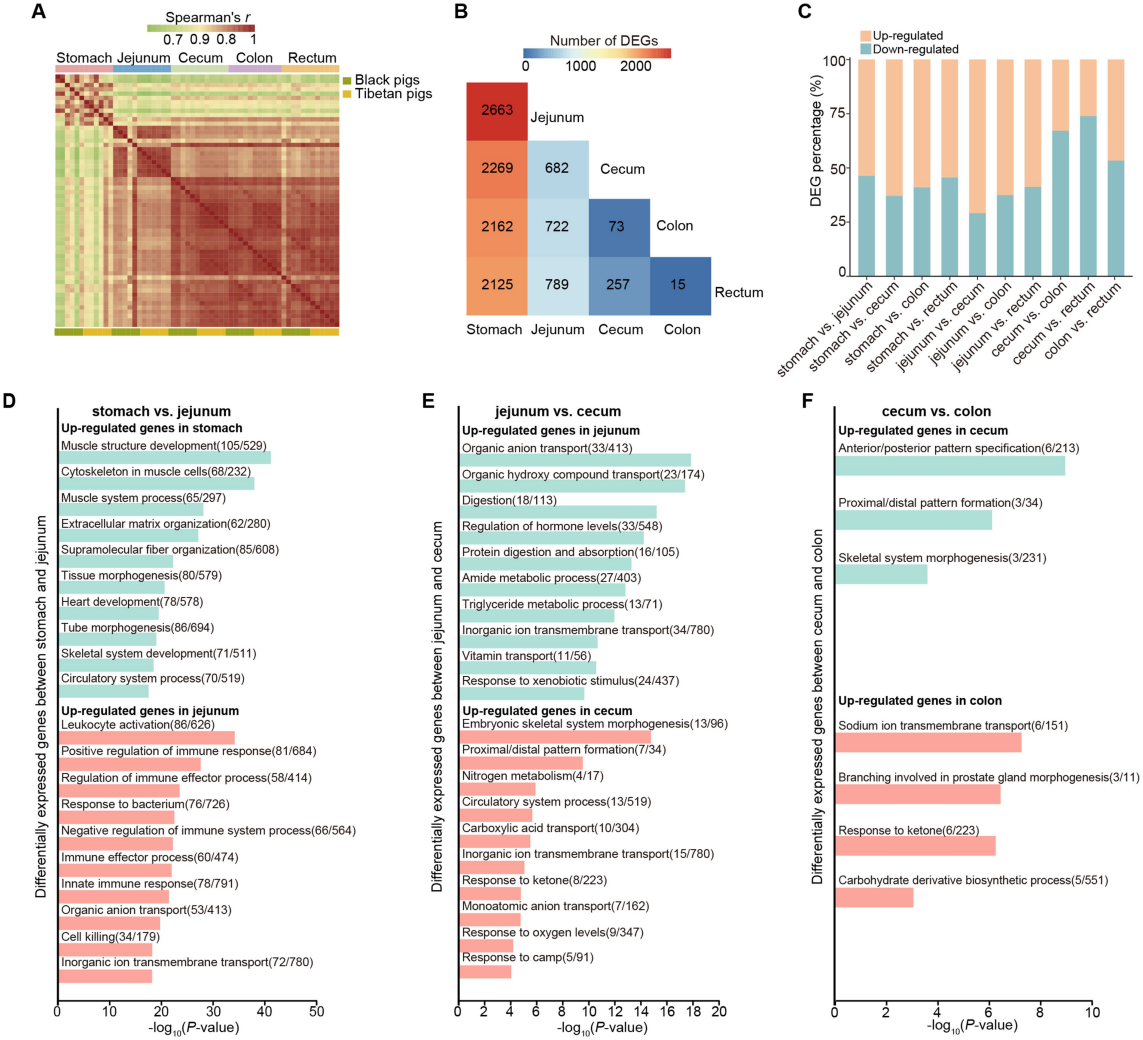
Comprehensive overview of the transcriptomes at five different gastrointestinal tract sites of pigs. **(A)** Spearman correlation heatmap showing the correlation between each RNA-seq sample. Color spectrum ranges from green, indicating weak correlation, to red, indicating strong correlation. **(B)** Heatmap of the numbers of pairwise comparisons of DEGs among the five different sites. **(C)** Bar plots showing the proportions of differentially upregulated (orange) and downregulated genes (blue) relative to the total number of DEGs in the gastrointestinal tract samples from each of two different sites (10 comparison groups in total). The exact numbers are shown in the corresponding positions in the plot. “up-regulated” refers to those genes with higher expression levels in the anterior tissue than in the posterior ones; “down-regulated” refers to those genes with lower expression levels in the anterior tissue than in the posterior ones. **(D–F)** Functional enrichment analysis of DEGs between different gastrointestinal tract sites.

We collected 59 samples from 5 gastrointestinal tract tissues (i.e., stomach, jejunum, cecum, colon and rectum) of Black pigs (live in low altitude, 461 m) and Tibetan pigs (live in high altitude, 3,750 m). To reveal the transcriptomic changes among the samples, we performed RNA-seq. A total of ~425 gigabases (Gb) high-quality data were obtained, with an average of 7.2 Gb per sample (Supplementary Table S1). Hierarchical clustering of the expression levels of these samples showed that the samples primarily clustered by tissue types and then by pig populations (Figure 1A). This finding indicates that the expression levels of genes are more diverged between different gastrointestinal tract tissues than between Black and Tibetan pigs.

We next compared the transcriptomic differences among the 5 tissues of the gastrointestinal tract. To better understand the general transcriptomic features across various gastrointestinal tissues, regardless of population, we merged the data of Black and Tibetan pigs together for each tissue. We performed differential expression analyses between pairwise comparisons of the five different gastrointestinal sites and counted the number of differentially expressed genes (DEGs) that differed significantly in each comparison (Figures 1B,C, Supplementary Figure S1). As expected, the remarkable differences were observed between stomach and other intestinal tissues (~2,304 DEGs detected in each comparison) (Figure 1B). Furthermore, we also found that the number of DEGs between the adjacent intestinal tissues decreases along the gut (from jejunum to rectum) (Figure 1B). This observation suggests an increasing similarity between these adjacent tissues.

We then explored the functional annotations for DEGs between adjacent intestinal tissues. We found that the genes upregulated in stomach (stomach vs. jejunum comparison) were enriched in the categories related to “muscle system” (Figure 1D, Supplementary Table S2). Nonetheless, the genes upregulated in the jejunum (stomach vs. jejunum) were mainly involved in the categories related to “immunity” (Figure 1D, Supplementary Table S3), which was in agreement with the intestine is the largest immune organ and with the largest number of immune cells in the body (25, 26). Moreover, in the comparison between jejunum and cecum, the genes upregulated in the jejunum were associated with the digestion and absorption of protein and lipid (Figure 1E, Supplementary Table S4), consistent with the jejunum is a major site for protein as well as for lipid digestion and absorption. For the genes upregulated in cecum (jejunum vs. cecum), they were mainly enriched in the categories associated with “circulatory system” and “ion transport” (Figure 1E, Supplementary Table S5), in line with the large intestine involved in regulation of the body fluid and electrolyte balance. Interestingly, although few DEGs were detected between cecum and colon (73 DEGs), many of them were enriched in pathways related to embryonic development and morphogenesis (e.g., “anterior/posterior pattern specification”) (Figure 1F, Supplementary Tables S6, S7). In particular, we observed 10 genes that belong to homeobox (HOX) superfamily, including *HOXA13*, *HOXC8*, *HOXB13*, *HOXC5*, *HOXC6*, *HOXC9*, *HOXC10*, *HOXC11*, *HOXD12*, *HOXD13*, were differentially expressed between cecum and colon. The HOX gene family are major regulators of animal development and morphogenesis (27, 28) and the changes in their expression level are correlate with the morphological variation of animals. Therefore, these differentially expressed HOX genes may be involved in the regulation of morphological differentiation between cecum and colon of pigs. Notably, we found that the genes related to the carbohydrate metabolism were more enriched in the colon relative to the cecum, suggesting the colon also play an important role in the carbohydrate metabolic process in pigs (Figure 1F, Supplementary Table S7). Finally, we only identified 15 DEGs between colon and rectum, implying the functional similarities between them.

### 3.2 Genes differentially expressed in five gastrointestinal tract tissues between high and low altitude pigs

**Figure 2:**
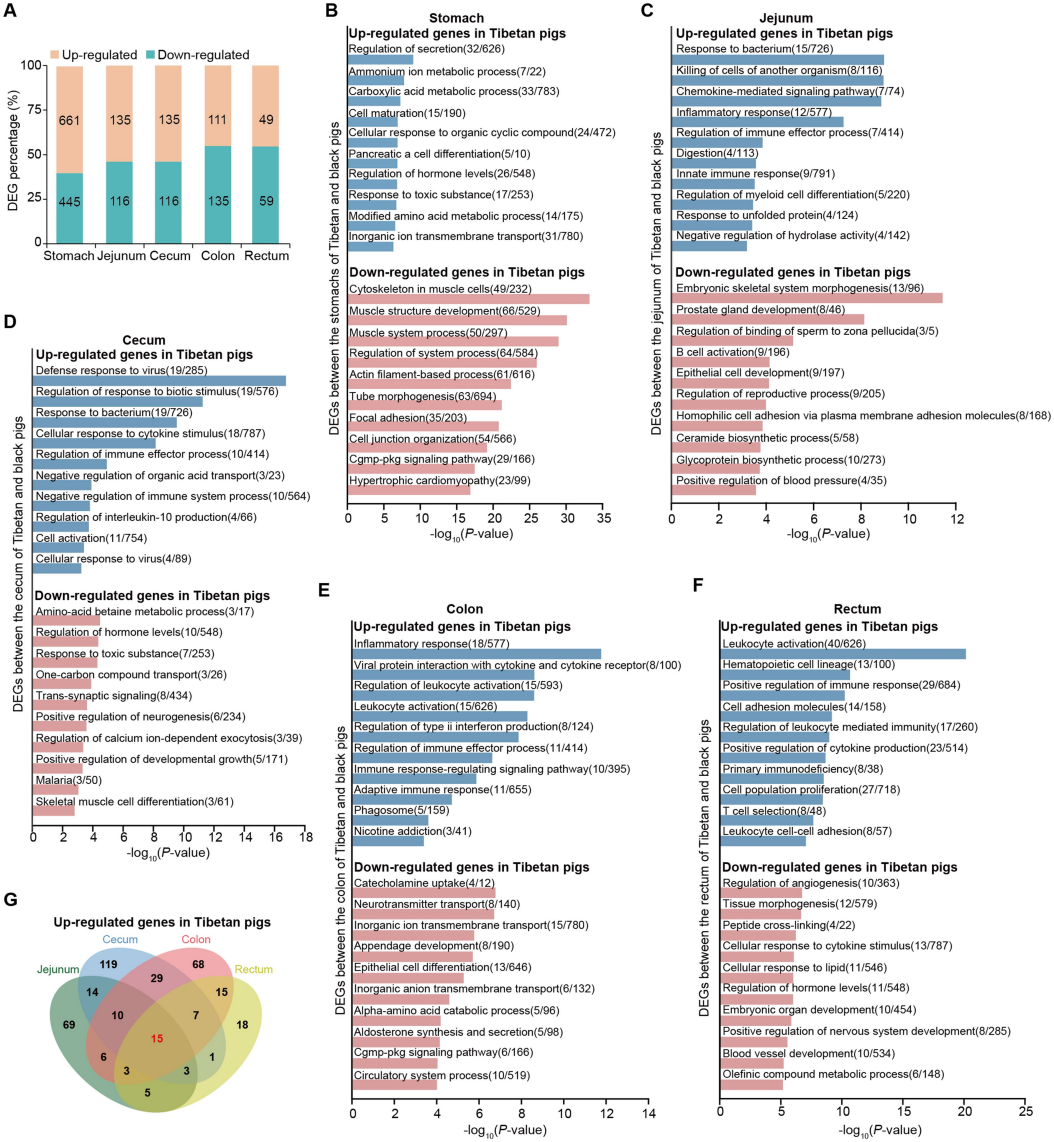
Transcriptomic differences in five gastrointestinal tract tissues between Tibetan pigs and Black pigs. **(A)** Bar plots showing the proportions of differentially upregulated (orange) and downregulated genes (blue) relative to the total number of DEGs between Tibetan and Black pigs at each site. Exact numbers are shown in the corresponding positions in the plot. “up-regulated” and “down-regulated” represent genes up-regulated or down-regulated in Tibetan pigs, respectively. **(B–F)** Functional enrichment analysis of DEGs between Tibetan pigs and Black pigs in stomach **(B)**, jejunum **(C)**, cecum **(D)**, colon **(E)** and rectum **(F)**. **(G)** Overlap among genes up-regulated in the Tibetan pigs.

To explore the gene expression changes at the same sites of the gastrointestinal tract between high and low altitude pigs, we first conducted a *t*-distributed stochastic neighbor embedding (*t*-SNE) analysis for each site in the Black and Tibetan pigs based on gene expression level. This analysis revealed a distinct separation between the different pig populations in the majority of examined tissues (Supplementary Figure 2), highlighting the transcriptional differences in the gastrointestinal tract between Black and Tibetan pigs. We next performed differential expression analysis for each gastrointestinal site between Tibetan and Black pigs. The most substantial difference was observed in the stomach, with 1,394 DEGs, whereas the rectum exhibited the least difference, only with 180 DEGs (Figure 2A). The remaining tissues demonstrated a relatively similar number of DEGs, ranging from 343 to 495 (Figure 2A, Supplementary Tables S8–S12).

We then explored the function of the identified genes that upregulated in the Tibetan pigs (i.e., high altitude pig population). Functional enrichment analysis revealed that the upregulated genes identified in the stomach were enriched in many categories associated with metabolic processes (e.g., “carboxylic acid metabolic process” and “amino acid metabolic process”) (Figure 2B, Supplementary Table S13). Previous studies have report that the high altitude coupled with hypoxia would lead to increased energy expenditure (29–31). Consequently, the observed alterations in gene expression within the stomach may have influenced the metabolic adaptations of pigs to high-altitude environments. Remarkably, we found that the immune-related pathways were enriched among the up-regulated genes in Tibetan pigs across all intestinal tissues (i.e., jejunum, cecum, colon and rectum) (Figures 2C–F, Supplementary Tables S14–S17). Supporting the Tibetan pigs are likely more resistant to disease (32). In addition, we observed that the genes with high expression levels in Tibetan pigs identified in the intestine were also overrepresented in categories associated with lipid metabolism-related pathways, including lipid transport, lipid catabolic process and lipid biosynthetic process (Figures 2C–F, Supplementary Tables S14–S17). Given that the previous research has identified the daily energy intake and proportion of fat supply in the Jiarong Tibetan population increase with altitude (33). These results suggest that lipid metabolism might provide an important source of energy supply for the Tibetan pigs, again supporting the transcriptional changes in the gastrointestinal tract may be involved in the high-altitude metabolic adaptation of pigs. We next ask which DEGs were shared among intestinal tissues. Among the identified 382 Tibetan pig up-regulated genes in the four intestinal tissues, 15 genes were tissue-shared (Figure 2G). Of which, seven genes with clear gene symbol in the Ensembl database (Release 114), including four gene involved in immune response [*DYNC1I1* (34), *RASGEF1A* (35), *ZBED2* (36) and *XCR1* (37, 38)] and one gene (*PLA2G2A*) related to lipid metabolism. It has previously been demonstrated that the suppression of *PLA2G2A* leads to a reduction in the expression of fatty acid synthase (*FASN*), an enzyme involved in *de novo* fatty acid synthesis (39). Consequently, we hypothesize that the upregulation of *PLA2G2A* in all four intestinal tissues of Tibetan pigs may facilitate their adaptation to high-altitude environments.

For the genes down-regulated in Tibetan pigs, we found that those in the stomach were mainly associated with development (e.g., “muscle structure development”) (Figure 2B, Supplementary Table S18). Similar results were also observed in the intestinal tissues (Figures 2C–E, Supplementary Tables S19–S21). These observations are consistent with the broader understanding that Tibetan pigs, characterized by their slow developmental pace, are classified as late-maturing breeds, whereas Black pigs demonstrate a more accelerated growth rate relative to Tibetan pigs. Additionally, our analysis revealed that the up-regulated genes identified in the intestinal tissues of Black pigs were significantly enriched in categories associated with metabolic processes, such as “one-carbon compound transport” and “alpha-amino acid catabolic process” (Figures 2C–F, Supplementary Tables S19–S22). This likely plays a crucial role in facilitating the rapid growth and development observed in the Black pigs. Among the identified Tibetan pig down-regulated genes in the four intestinal tissues, eight genes (*NTN5*, *CNDP1*, *TFR2*, *DMP1*, *SCD5*, *SLC13A4*, *TKTL2*, *AFMID*) were found to be shared across these tissues. These genes are primarily involved in metabolic processes, including protein metabolism, fatty acid metabolism, and L-tryptophan catabolism.

### 3.3 Genes differentially expressed in triceps brachii muscle between two pig population

Previously, it was reported that the intestine may also be involved in regulating mammalian skeletal muscle function and metabolism (40, 41). To reveal the changes in skeletal muscle function between the Black and Tibetan pigs, we sampled the triceps brachii muscles from each of these two pig populations (*n* = 6) and performed RNA-seq. In total, 144.61 million and 144.39 million reads were obtained from the triceps brachii muscle of the Tibetan and Black pigs, respectively. After filtering out the low-quality sequences, 43.38 and 43.32 Gb were obtained from the Tibetan and Black pigs, respectively, for further analysis (Supplementary Table S1).

**Figure 3:**
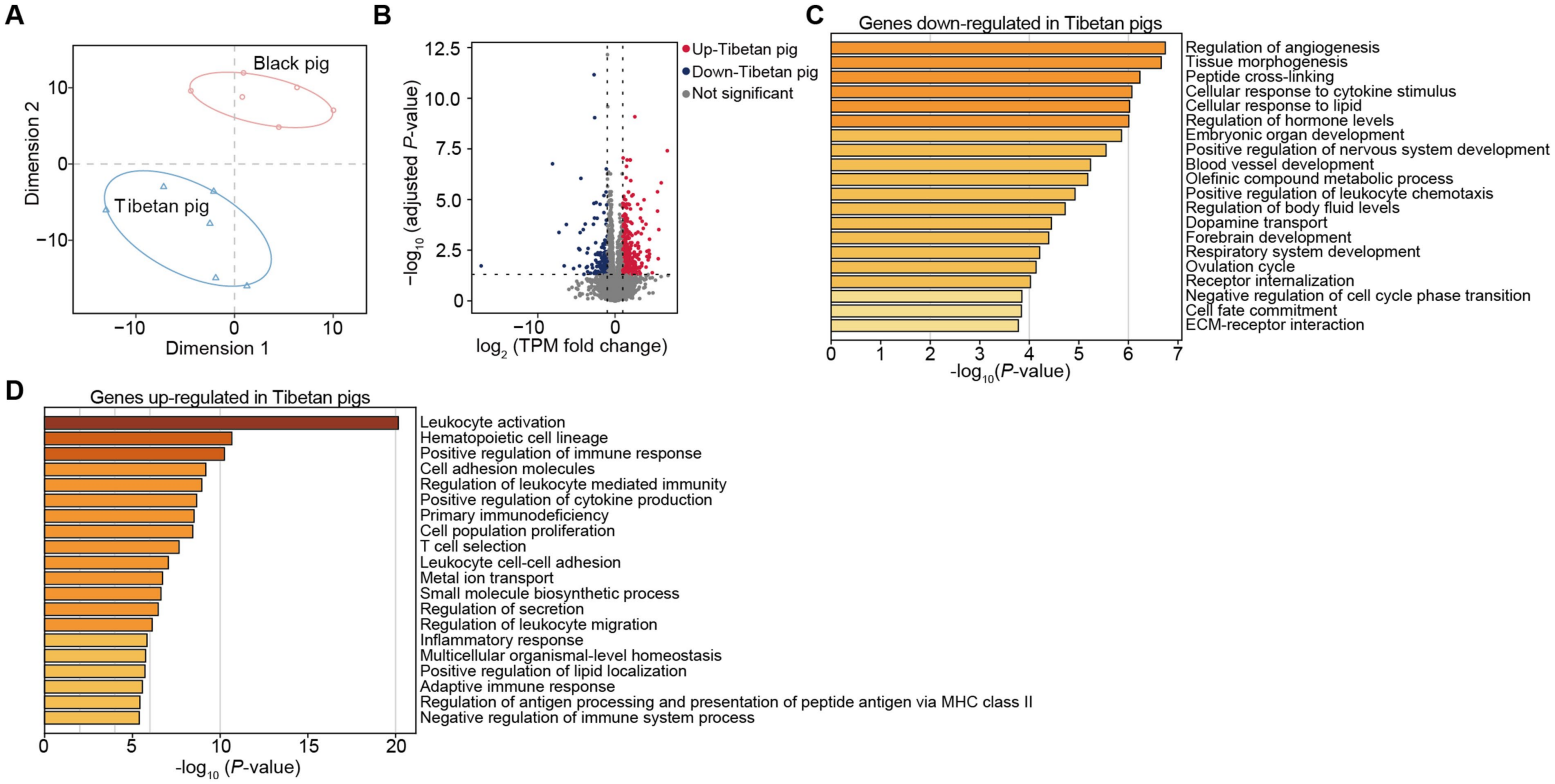
Transcriptomic differences in triceps brachii muscles between Tibetan pigs and Black pigs. **(A)** t-distributed stochastic neighbor embedding (t-SNE) showing the separation of samples. The distances between samples represent the similarity between samples. **(B)** Volcano map shows the differentially expressed genes of the triceps brachii muscles in Tibetan and Black pigs. Red (up-regulated genes in Tibetan pigs) and blue dots (down-regulated genes in Tibetan pigs) indicate differentially expressed genes, and grey dots indicate similarly expressed genes. **(C,D)** Functional enrichment analysis of DEGs up-regulated in Black pigs **(C)** and Tibetan pigs **(D)**, respectively.

A *t*-SNE analysis showed significantly different expression profiles in the Tibetan and Black pigs (Figure 3A). The analysis detected 535 significant DEGs (|log_2_fold change| ≥ 1, Benjamini Hochberg corrected *p* value<0.05) (Figure 3B). Similar to the above findings in the gastrointestinal tissues, functional enrichment analysis showed that the genes down-regulated in Tibetan pigs were mainly related to growth and development (e.g., “embryonic organ development”) (Figure 3C, Supplementary Table S23). In contrast, the genes up-regulated in Tibetan pigs were mainly related to the immune function, including “leukocyte activation” and “positive regulation of immune response” (Figure 3D, Supplementary Table S24). Results again supported the high disease resistance of Tibetan pigs. Furthermore, the categories associated with fatty acids biosynthesis and metabolism (e.g., “positive regulation of lipid localization”, “unsaturated fatty acid biosynthetic process” and “fatty acid biosynthetic process”) were also overrepresented in the up-regulated genes in Tibetan pigs (Figure 3D, Supplementary Table S23), implying there might be some differences in the fatty acid composition between the two pig populations.

### 3.4 Amino acids, fatty acids, and flavor characteristics of triceps brachii in Tibetan and Black pig

**Figure 4:**
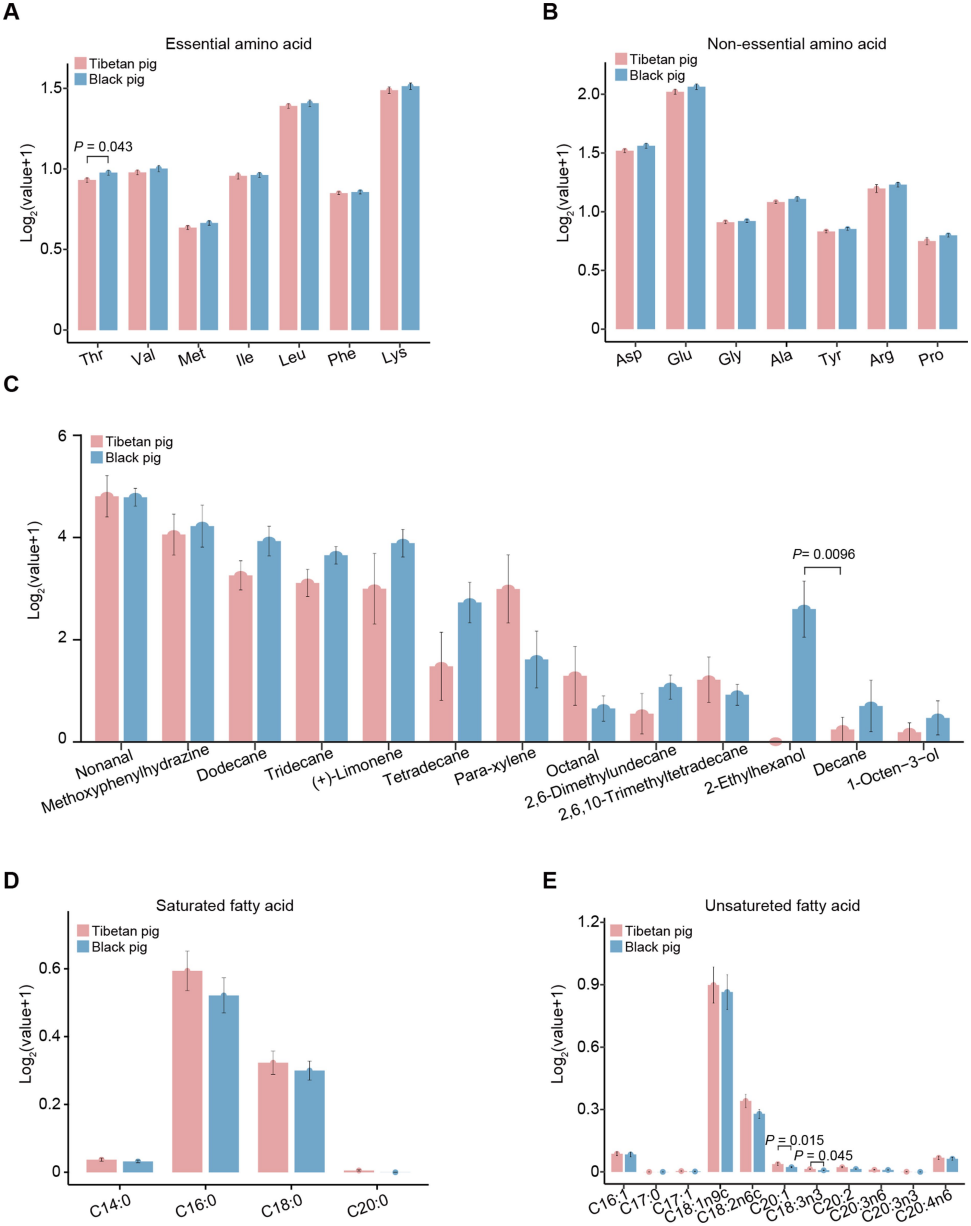
The contents of amino acid, fatty acid, and flavor characteristics of triceps brachii in Tibetan and Black pigs. **(A)** Essential amino acids in the triceps brachii of Tibetan and Black pigs (*n* = 6 for each population). Thr, threonine; Val, valine; Met, methionine; Ile, isoleucine; Leu, leucine; Phe, phenylalanine; Lys, lysine. **(B)** Nonessential amino acid contents in Tibetan pig and Black pig (*n* = 6 for each population). Asp, aspartic acid; Glu, glutamic acid; Gly, glycine; Ala, alanine; Tyr, tyrosine; Arg, arginine; Pro, proline. **(C)** Flavor characteristics of the Tibetan and Black pigs (*n* = 6 for each population). **(D)** Saturated fatty acid contents of the triceps brachii muscles from the Tibetan and Black pigs (*n* = 6 for each population). **(E)** Unsaturated fatty acid contents of the triceps brachii in the Tibetan and Black pigs (*n* = 6 for each population).

To further elucidate the metabolic differences between the triceps brachii muscles of Tibetan and Black pigs, an analysis was conducted to measure the abundance of amino acids, fatty acids, and flavor characteristics, which are also indicative of meat quality. The results detailing the amino acid, fatty acid, and flavor profiles of the triceps brachii samples are presented in Figure 4. Among the 14 amino acids analyzed, only threonine (Thr) showed a significant difference (*p* = 0.043), with its content being notably higher in the Black pig compared to the Tibetan pig. Regarding the 13 flavor substances examined, the content of 2-ethylhexanol was significantly higher (*p* = 0.0096) in the Black pig than in the Tibetan pig (Figure 4C). On the contrary, in the analysis of 15 fatty acids, the contents of C20:1 (*p* = 0.015) and C18:3n6 (*p* = 0.045) were significantly higher in the Tibetan pigs than in the Black pigs (Figures 4D,E). This is consistent with the above finding that the genes with high expression level in Tibetan pigs were closely related to fatty acids biosynthesis and metabolism. C20:1, also known as cis-11-eicosenoic acid and gondoic acid, is involved in the anti-inflammatory responses and the inhibition of reactive oxygen species production (42). Moreover, C18:3n6 (gamma linolenic acid) has also been reported as an anti-inflammatory fatty acid (43). Therefore, the increased level of C20:1 and C18:3n6 in the triceps brachii of Tibetan pigs might contribute to reduce inflammation and oxidative damage caused by hypoxia.

### 3.5 Correlation analysis of gastrointestinal gene expression and metabolite abundance of triceps brachii

**Figure 5:**
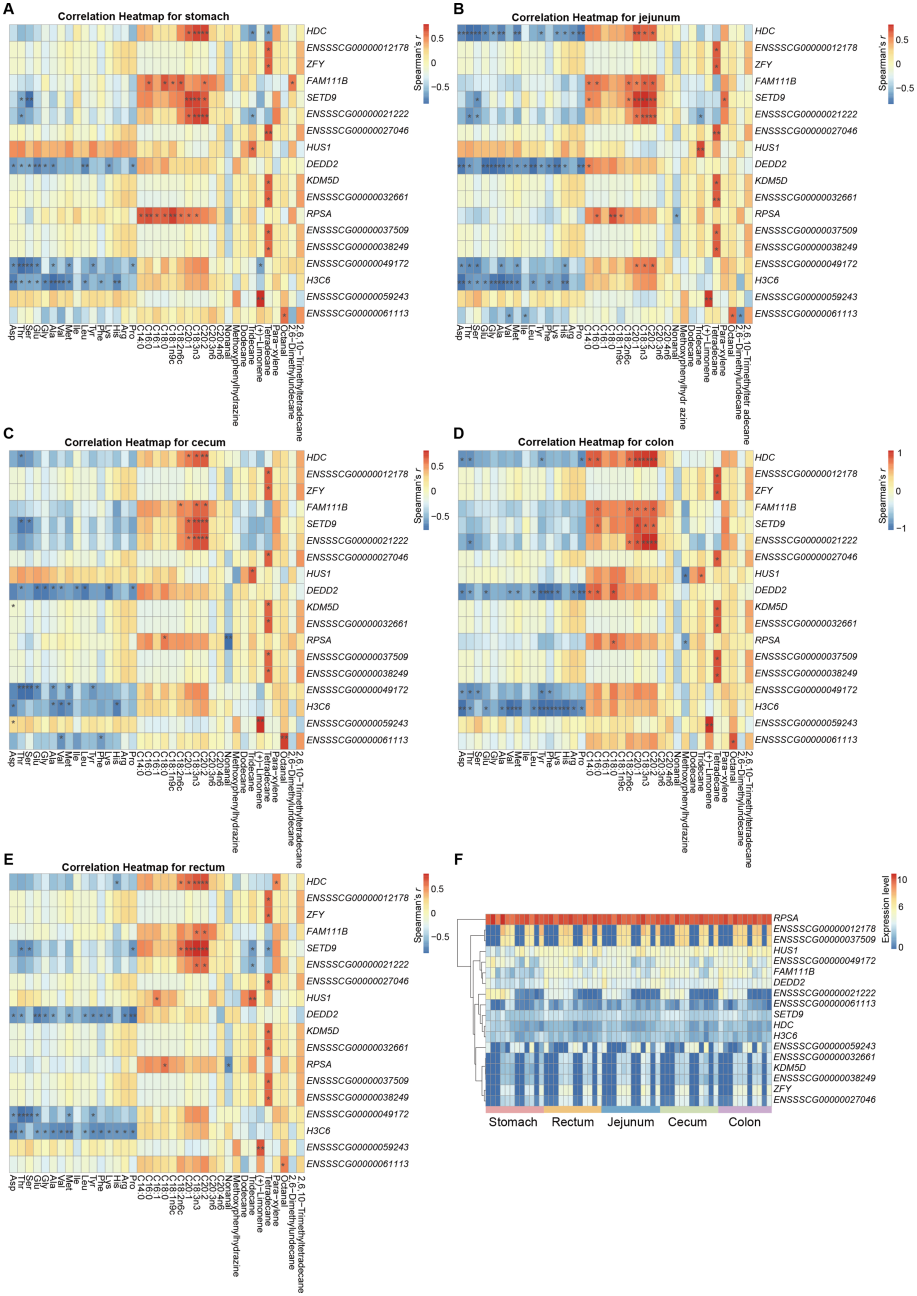
Genes significantly associated with metabolite abundance at all five gastrointestinal sites in both pig populations. **(A–E)** Heatmaps showing the correlation between gene expression at each site and metabolite abundance. **(F)** Relative expression of 18 genes that significantly associated with metabolite abundance.

To explore the potential associations between gene expression in various gastrointestinal regions and metabolite abundance in the triceps brachii in pigs, we integrated the data from Black and Tibetan pigs for each tissue type to calculate the Spearman’s correlation coefficients for the expression of each gene and specific metabolite abundance (i.e., amino acid, fatty acid, and flavor characteristics) within each tissue. We identified 18 genes (*HDC*, *ENSSSCG00000012178*, *ZFY*, *FAM111B*, *SETD9*, *ENSSSCG00000021222*, *ENSSSCG00000027046*, *HUS1*, *DEDD2*, *KDM5D*, *ENSSSCG00000032661*, *RPSA*, *ENSSSCG00000037509*, *ENSSSCG00000038249*, *ENSSSCG00000049172*, *H3C6*, *ENSSSCG00000059243*, and *ENSSSCG00000061113*) significantly associated with the metabolite abundance at all five gastrointestinal sites (Benjamini Hochberg corrected *p* value<0.05) (Figure 5). Of which, 9 genes (*KDM5D*, *ZFY*, *HUS1*, *RPSA*, *DEDD2*, *H3C6*, *FAM111B*, *SETD9*, and *HDC*) have been reported to be associated with meat quality traits in previous studies. Typically, previous analysis implicated *HUS1* was associated with the intramuscular fat content in pigs (44). We consistently observed a significant correlation between the expression of *HUS1* in the gastrointestinal tract and the presence of the unsaturated fatty acid C16:1, as well as the flavor compound tridecane, thereby reinforcing the reliability of our analysis. In another example, the *HDC* gene, which encodes a rate-limiting enzyme in the histamine synthesis pathway, exhibited a significant positive correlation between its expression in the gastrointestinal tract and the abundance of the unsaturated fatty acid C20:1, known for its beneficial effects on human health (45). The expression levels of the *HDC* gene were significantly higher in the gastrointestinal tissues of Tibetan pigs compared to Black pigs, which was also confirmed by qPCR validation (Supplementary Figure 3). Correspondingly, the abundance of C20:1 was also significantly greater in the triceps brachii of Tibetan pigs than in Black pigs. Previous research has demonstrated that histamine infusion can increase the levels of free fatty acids in plasma. Therefore, the elevated expression of the *HDC* gene in the gastrointestinal tissues of Tibetan pigs may contribute to the accumulation of C20:1 in their triceps brachii, thereby enhancing the nutritional quality of Tibetan pig meat. Consequently, these genes could serve as potential candidates for genetic breeding programs aimed at enhancing meat quality in pigs.

Specifically, the amino acid contents mainly correlated negatively with the expression of genes *DEDD2*, *H3C6*, *ENSSSCG00000049172*, *HDC*, and *SETD9*. The fatty acid contents mainly correlated positively with expression of genes *ENSSSCG00000021222*, *HDC*, *SETD9*, *HUS1*, and *RPSA*. The expression of *KDM5D*, *ZFY*, and several novel genes (*ENSSSCG00000012178*, *ENSSSCG00000027046*, *ENSSSCG00000032661*, *ENSSSCG00000037509*, and *ENSSSCG00000038249*) correlated positively with the tetradecane (flavor substance) content (Figures 5A–E). Notably, we found that the expression of these genes was primarily correlated with the fatty acid content and amino acid content of the triceps brachii in opposite ways (Figures 5A–E). Therefore, it will be important to accommodate these trends to enhance the quality of meat, and to identify genes whose expression correlates either positively or negatively with the vast majority of important meat quality traits.

## 4 Conclusion

In this study, we conducted a comparative analysis of gene expression across five gastrointestinal regions and the triceps brachii muscles between Tibetan pigs and Black pigs. Genes exhibiting elevated expression levels in Tibetan pigs, relative to Black pigs, were predominantly associated with immune response and energy metabolism (particularly lipid metabolism). Furthermore, we identified 18 genes within gastrointestinal tract tissues whose expression levels demonstrated significant correlations with the amino acid, fatty acid, and flavor profiles of the triceps brachii muscles. These findings enhance our understanding of the role of gastrointestinal tract tissues in the high-altitude adaptation of Tibetan pigs.

## Data Availability

The raw RNA-seq data of gastrointestinal tissues of pigs generated in this study are available in Sequence Read Archive (SRA) under BioProject “PRJNA1192511”. The raw RNA-seq data of triceps brachii of pigs are available in SRA under BioProject “PRJNA1192560”. The names of the accession numbers can be found in the Supplementary material.
