# Supplementary material for: Transcriptional insights into gastrointestinal adaptations in pigs to high altitude
Source: Front Vet Sci. 2025 Dec 4;12:1723710. doi: 10.3389/fvets.2025.1723710 (PMC12711528; doi:10.3389/fvets.2025.1723710)
Supplement: Supplementary file 1 [file Data_Sheet_1.DOCX]

Supplementary Figures


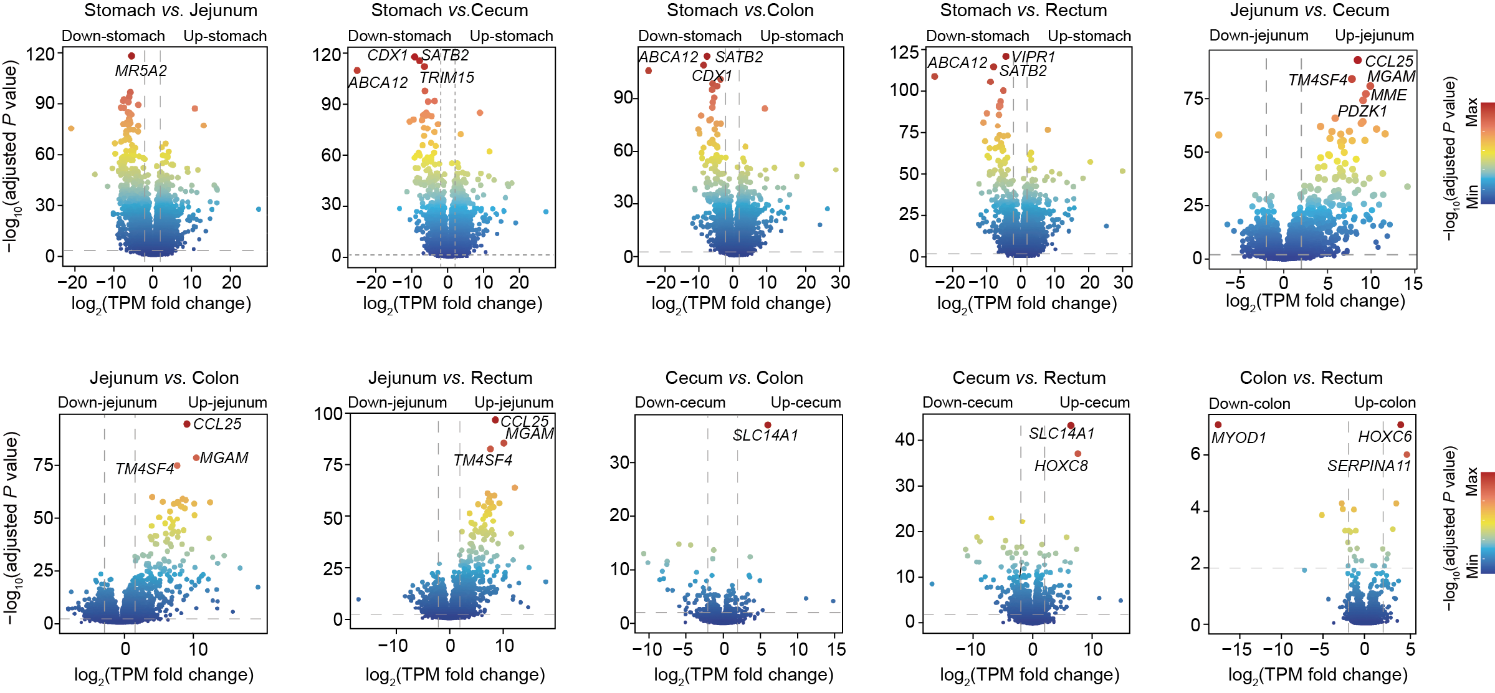


**Supplementary Figure 1.** Volcano plot of differentially expressed genes between different gastrointestinal sites.


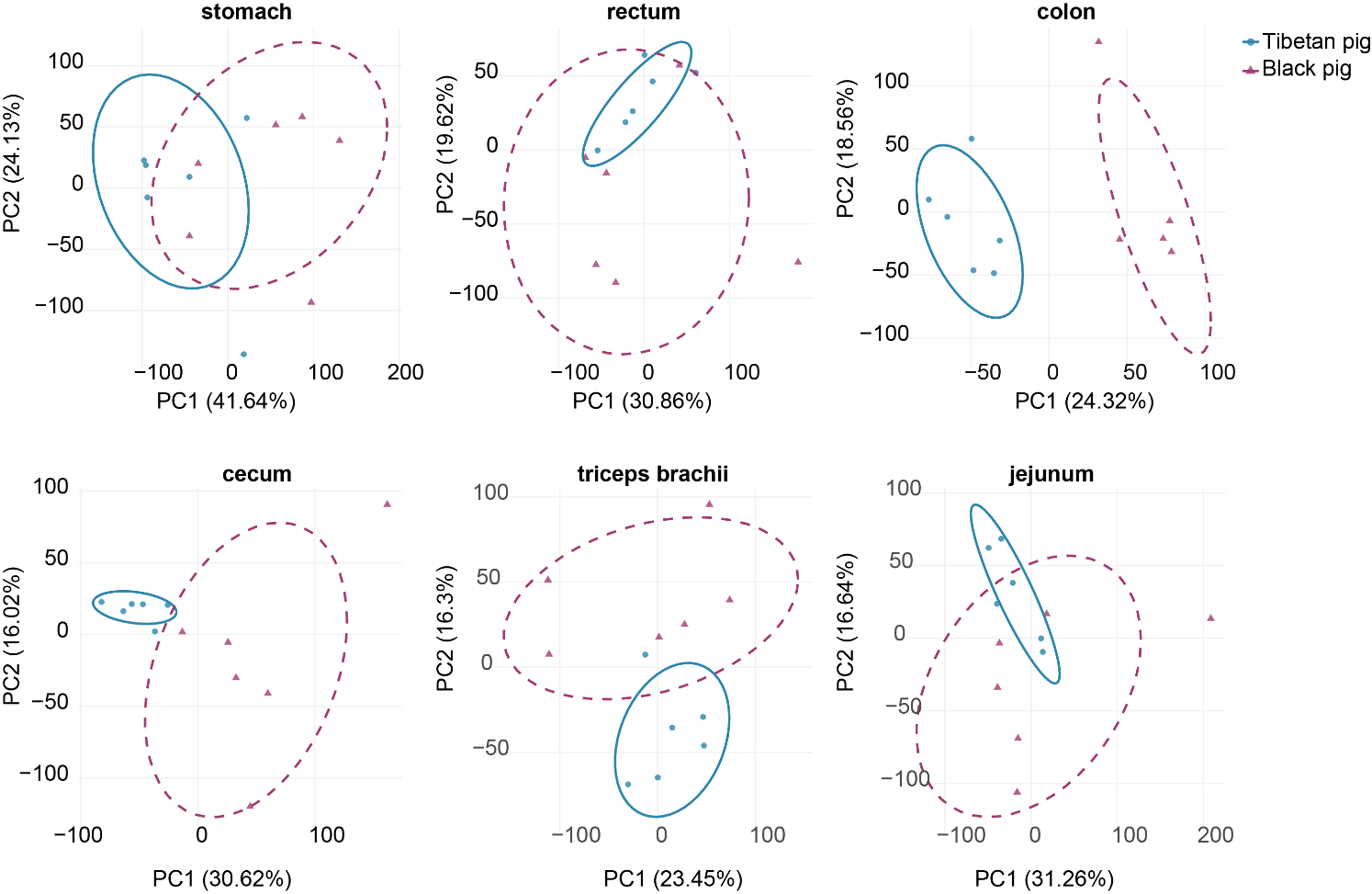


**Supplementary Figure 2.** The PCA plots of the Tibetan pig (blue) and Black pig (purple) samples based on the gene expression.


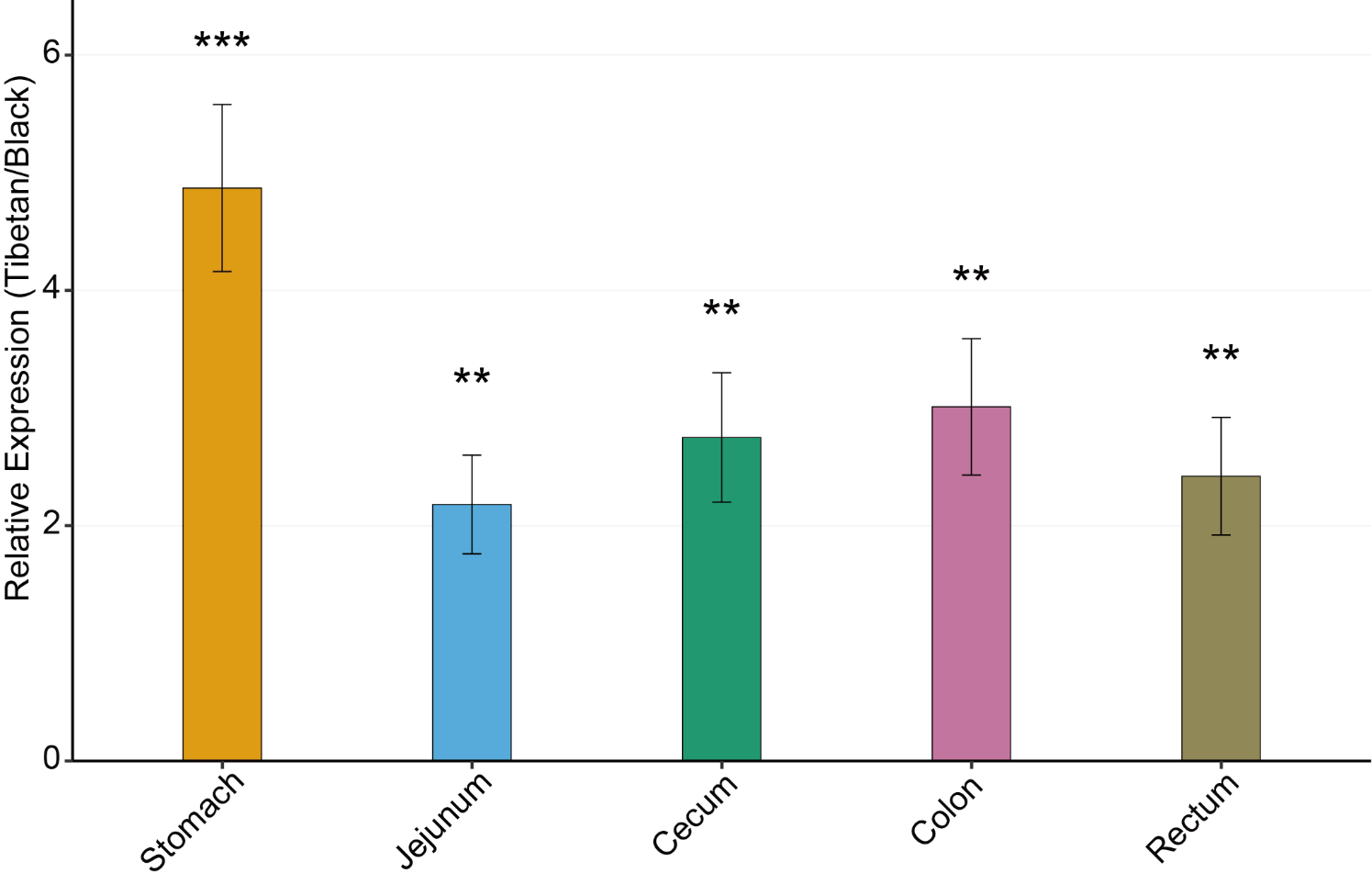


**Supplementary Figure 3.** qPCR validation for *HDC* gene expression in Tibetan and Black pig tissues. Data are shown as mean ± SD. ** *P* ≤0.01, *** *P* ≤0.001
